# Unraveling the adsorption potential of Zr dithiol (MOF-DSH) through experimentation and neural network modeling†

**DOI:** 10.1039/d5ra00002e

**Published:** 2025-04-15

**Authors:** Nitin Gumber, Buddhadev Kanrar, Jaspreet Singh, Jitendra Bahadur, Rajesh V. Pai

**Affiliations:** a Fuel Chemistry Division, Bhabha Atomic Research Centre Mumbai 400085 India rajeshvp@barc.gov.in; b Technical Physics Division, Bhabha Atomic Research Centre Mumbai 400085 India; c Solid State Physics Division, Bhabha Atomic Research Centre Mumbai 400085 India; d Homi Bhabha National Institute Anushaktinagar Mumbai 400085 India

## Abstract

In this study, an aqueous-stable metal–organic framework with two thiol groups was synthesized using Zr as the metal centre and dimercaptosuccinic acid as the ligand through a conventional heating method for the removal of Cd(ii) from aqueous solution. Different characterization tools, including XRD, FT-IR, BET, SEM, TGA, and XPS, were employed. XRD results showed a characteristic pattern of a hexa-cluster, which was in agreement with the simulated MOF-801, and the corresponding vibrational peaks were observed in the FT-IR spectra. The synthesized MOF was thermally stable up to 300 °C, as demonstrated by TGA, and exhibited a specific surface area of 290 m^2^ g^−1^. Cadmium adsorption studies performed at different pH values showed a maximum adsorption capacity of 91.5 mg g^−1^ at pH 6. The adsorption behavior was well described by the Langmuir model and the pseudo-second order (PSO) kinetics, confirming the involvement of a monolayer with chemisorption as the dominant mode of adsorption. The synthesized MOF could be reused at least 4 times while retaining ∼80% of its initial adsorption capacity. FT-IR, XPS, and pH studies after Cd(ii) adsorption revealed that the predominant mode of interaction of Cd(ii) with the MOF is an ion-exchange mechanism. An artificial neural network-based (ANN) methodology was employed to model the adsorption capacity of Cd(ii) and predict the adsorption capacity as a function of Cd(ii) concentration, time of contact, and pH of the medium. The model demonstrated excellent results, with an average error of 2.3% and precision of 3.0%. The outcomes of these studies were consistent with the experimental results.

## Abstract 2

Efficient removal of Cd(ii) using thiol based Zr metal organic framework: experimental and neural network modeling.

## Graphical abstract

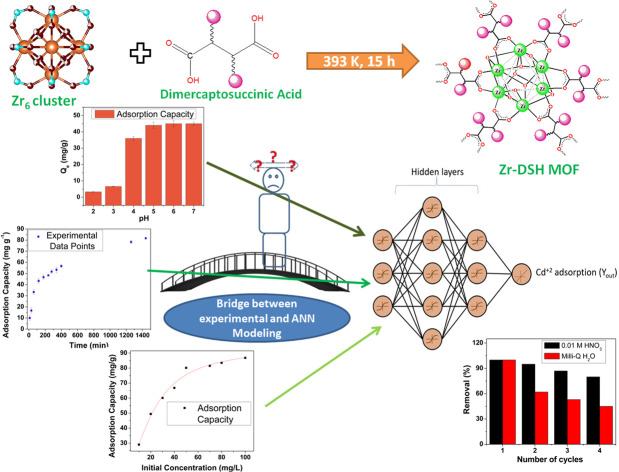

## Introduction

Fast industrialization, augmented agricultural production and swift urbanization have resulted in an upsurge in water pollution.^1^ In contrast to dyes and other polymers, heavy metal ions, such as Cd, Hg, and others, do not degrade over time. It is well known that cadmium is far more hazardous than mercury.^2^ Through various pathways, including drinking water and agriculture, the presence of Cd in aqueous streams can spread through the ecosystem. Cadmium accumulates in the body after consumption, especially in the kidneys, where it causes malfunctions. Significant consumption of Cd is known to be extremely toxic and to have a number of negative effects, the most common of which are cancer, osteomalacia, anemia, and neurological problems.^3^ The textile, cement, and fertilizer sectors are the primary sources of Cd emissions.^4^ Different organizations around the world have already included Cd in their red/black lists, and the World Health Organization (WHO) has recommended a maximum permissible intake limit of 5 μg L^−1^ for Cd in drinking water.^5,6^ Thus, it is imperative to remove Cd from various aqueous streams to ensure the sustainability of life forms and prevent environmental damage.

Currently, electrochemical,^7^ precipitation,^8^ membrane,^9^ crystallization,^10^ adsorption,^11^*etc.* are employed for the remediation of Cd(ii) from aqueous solutions. Adsorption using solid-state adsorbents is unique among the aforementioned methods due to its simplicity, low energy consumption, and minimal generation of secondary waste. The structural attributes of metal–organic frameworks (MOFs) significantly augment their effectiveness in environmental remediation by adsorbing heavy metal ions from aqueous solutions, and they have attracted significant attention from the research community engaged in this field.^12^ MOFs are three-dimensional porous materials with regularly spaced holes that are kept together by inorganic metal ions and organic ligands.^13^ MOFs have a wide range of uses, including adsorption, drug delivery and catalysis.^14–16^

Also, these materials can be used as stimuli-responsive nanomaterials, offering significant potential for improving water treatment, as reported in literature.^17^ In addition to their ease of functionalization to induce selectivity for a particular metal ion, their large surface area and pore volume make them suitable candidates for the adsorption of heavy metal ions.^18,19^

Aqueous stability is the main factor influencing the use of an MOF to be used for the adsorption of heavy metal ions. Zr-based carboxylate MOFs are known to be very stable due to the hard–hard interactions between their inorganic and organic centers. Dimercaptosuccinic acid was utilized as a linker having two carboxylate groups for chelation with Zr and 2 thiol groups would still be available for the adsorption of metal ions. Given that –SH groups are soft bases, they can be utilized for the adsorption of soft metal ions such as Cd^2+^. The introduction of thiol groups in the structure of MOFs have resulted in an increased adsorption capacity for Cd(ii) compared to unfunctionalized MOFs previously. Using the same principles, Park *et al.*^20^ synthesized a thiol-functionalized activated carbon-based adsorbent and observed an adsorption capacity of ∼27.7 mg g^−1^ at pH 7. Similarly, Wu *et al.*^21^ synthesized thiol-functionalized silica, which was further coated on MOF-5, and obtained an astonishing adsorption capacity of 98.03 mg g^−1^. Ma *et al.*^22^ synthesized a sulfonic acid-functionalized Cu BTC-based MOF-808 with an adsorption capacity of ∼88.7 mg g^−1^. Zhang *et al.*^23^ synthesized a thiol-derived UiO-66 and found the maximum adsorption capacity of ∼77.42 mg g^−1^ at pH 6.

Generally, the adsorption process is used in industry such as water treatment and textile dying. The method for process optimization involves extensive experimental methodology, which is very tedious and time-consuming. On the contrary, computational simulation provides a flawless methodology to predict the adsorption process for various adsorbents. These simulations include molecular dynamics (MD), Monte Carlo (MC) simulation, quantum mechanical (QM) calculation, and machine learning (ML) methods.^24–26^ These methods deal with the detailed molecular interactions involved in the adsorption mechanism that are important for designing efficient adsorption systems. For example, molecular dynamics deals with the molecular arrangement and interaction energy with adsorbent materials.^25^ In the QM calculation, specifically DFT-based calculation, the adsorption mechanism is understood in-terms of molecular structure and reactivity descriptor and identified as interactions such as hydrogen bonding and electrostatic forces and stable adsorption configurations. MC samples the molecular configuration for simulating the equilibrium properties and adsorption energies.^26^ The ML methodology offers a highly efficient predictive model for optimizing the adsorption behavior in a cost-effective manner. The modes of operation for ML algorithms are handling big data, learning from existing experimental data and predicting unknown experimental outcomes.^27–29^ The model learns from the input variables such as concentration, pH, temperature, and adsorbent properties such as surface area and pore size as a function of adsorption capacity. The aim of ML-based algorithms is to determine the correlation between input and output variable. Unlike other ML-based techniques such as support vector regression (SVR), random forest, and *K*-nearest neighbor (KNN), artificial neural network (ANN) replicates the brain structure with inter connected layers of neurons. The purpose of interconnected layers is to follow the non-linear relationship between the input and output and adjust the connection between neurons. An ANN-based model can extract information from noisy and high variance data without knowing the underlying mathematical model. The important advantage of the ANN-based methodology is the robustness of the process in analyzing the data for quantitative information extraction. Hence, the major advantage of ML is the identification of the complex, non-linear relationships between input and output parameters that might be missed by other traditional methods.^24^ However, the performance of the ML method heavily relies on the size and quality of the training dataset.

Hence, herein, an ANN-based model was proposed to predict Cd(ii) adsorption using a thiol-based Zr MOF for the first time. In the outset, the obtained data was used to train the neural network-based algorithm, which was further validated and applied to the obtained experimental results.

The synthesis of thiol-based MOFs typically involves the use of complicated ligands that necessitate several stages. Furthermore, because of the conjugating effect, the presence of –SH groups on the aromatic ring lowers their activity. Cd(ii) is a neglected element, whereas the majority of documented thiol-based MOFs focus on removing mercury ions from aqueous solutions. Thus, the current work aimed to bridge the research gap on this topic. For convenience, the synthesized MOF is named MOF-DSH hereon. Its successful synthesis was established through characterization techniques such as X-ray diffraction (XRD), Fourier transform infrared spectroscopy (FT-IR), Brunauer–Emmett–Teller (BET), thermogravimetric analysis (TGA), and scanning electron microscopy (SEM). Different adsorption studies were performed, which included pH variation, adsorption kinetics, adsorption isotherms, reusability and selectivity. The state-of-art AI-based modeling was carried out using the data obtained in the adsorption process and a neural network-based modeling was built. The data was simulated as a function of initial concentration, pH of the medium and time using the ANN-based method, as described in detail herein. In the final part, a probable mechanistic view of adsorption is presented using techniques such as XRD, FT-IR and XPS (X-ray photoelectron spectroscopy).

## Experimental details

### Chemicals

The chemicals used in the study were procured from different sources and used as received without further purification unless specifically mentioned. Milli-Q water was used for the preparation of all solutions and necessary dilutions. For the synthesis of MOF, *meso*-2,3-dimercaptosuccinic acid (DSH, 98%) and zirconium tetrachloride anhydrous (ZrCl_4_) were procured from Sigma-Aldrich. Formic acid (HCOOH) was acquired from Fine Chemicals and ethanol (99.9%) was obtained from commercial alcohols. For the adsorption experiments, cadmium nitrate tetrahydrate (Cd(NO_3_)_2_)·4H_2_O was procured from SRL chemicals. Nitrate compounds of cobalt, strontium, silver, calcium and potassium were used for the selectivity experiments.

### Synthesis of MOF-DSH

MOF-DSH was synthesized according to a literature report with slight modifications.^30^ Equimolar amounts of ZrCl_4_ and dimercaptosuccinic acid were added to 225 equivalents of water and 2 equivalents of formic acid in a 4 dram glass vial with a screw cap. The mixture was sonicated for ∼15 min and heated at 393 K for 15 h. Subsequently, the product formed was washed with water and ethanol thrice using 20 mL solvent for 30 min in each cycle and dried at 373 K in an oven.

### Characterization

After synthesizing MOF-DSH, it was characterized thoroughly using diverse techniques including XRD, FT-IR, BET, TGA, SEM, and XPS. The crystal structure was determined using a Rigaku Miniflex XRD instrument using a Cu-Kα beam and attaching an Ni filter to remove Kβ residue. The 2*θ* angle of diffraction, was varied from 5° to 50° at a scan rate of 1° min^−1^. The vibrational spectra in the wavenumber range of 500–4000 cm^−1^ were recorded on a Bruker FT-IR table-top spectrophotometer at a resolution of 4 cm^−1^. The specific surface area of MOF was determined through gas adsorption experiments employing N_2_ gas. The sample was kept at liquid nitrogen temperature throughout the experiment and adsorption–desorption isotherms were obtained under a dynamic N_2_ atmosphere using a Sorptomatic 1990 analyzer. Prior to carrying out the surface area experiments, the sample was heated at 373 K for 6 h under high vacuum. The thermal stability of MOF was evaluated from RT to 873 K using a Mettler thermoanalyzer using ∼100 mg of sample. To probe the morphological details of MOF-DSH, an SNE-4500M mini SEM model was used. A very thin layer (Å level) of gold was coated on the sample to enhance its electrical conductivity and to eliminate charge accumulation. To investigate the binding energy of different elements, BL-9 located at Raja Ramanna Centre for Advanced Technology (RRCAT), Indore was used and the detailed XPS instrumentation is presented in the ESI.†

### Cadmium adsorption experiments

All Cd(ii) adsorption experiments were conducted at 25 °C ± 5 °C. A Cd(ii) stock solution with a concentration of ∼500 mg L^−1^ was prepared by dissolving a pre-determined amount of Cd(NO_3_)_2_·4H_2_O in Milli-Q water. It was analyzed using total X-ray fluorescence (TXRF) and the methodology is described in the ESI.† For the pH experiments, 3 mg of MOF-DSH was added to 10 mL of 50 mg per L Cd(ii) solution and equilibrated for 5 h using a shaker. The pH of the solution was varied from 2–7 by adding a negligible amount of 0.1 M HNO_3_ and 0.1 M NaOH. Further, adsorption kinetics studies were undertaken by fixing the pH at 6 and equilibrating the MOF-Cd(ii) solution for 24 h. The supernatant solution was analyzed at different intervals to assess the rate of adsorption. To perform the adsorption isotherm experiments, the initial concentration of Cd(ii) was varied in the range of 10–100 mg L^−1^ and the mixture was equilibrated for 24 h. For the selectivity studies, equimolar amounts of different metal ions were prepared and MOF was equilibrated for 24 h. The reusability of MOF was evaluated through multiple adsorption–desorption cycles and the adsorbed Cd(ii) was eluted using different eluting mediums, as mentioned in the reusability section. In all the above-mentioned experiments, centrifugation was carried post-adsorption and the supernatant was analyzed for Cd(ii) concentration using TXRF. The sample spectra obtained using TXRF were fitted using the PyMCA software, as depicted as ESI Fig. S1.† The adsorption capacity (*q*_e_) was evaluated using eqn (1), as follows:1
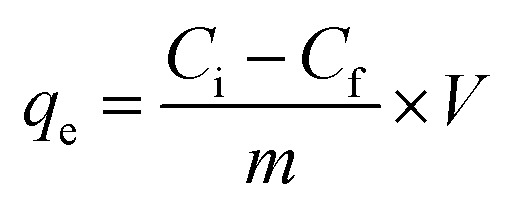
where *q*_e_ represents the equilibrium adsorption capacity in mg g^−1^. *C*_i_ and *C*_f_ represent the Cd(ii) concentration before and after adsorption in mg L^−1^, respectively. *V* and *m* denote the volume of Cd(ii) solution in mL and mass in mg of MOF-DSH utilized for adsorption, respectively.

### Computational and architectural details for artificial neural network

In the presently developed computational modeling, the adsorption data was simulated using an ANN-based method. This method involved training the data to obtain weight and bias parameters for the neural network.^31,32^ A schematic diagram of ANN is presented in ESI Fig. S2.†

Herein, several cadmium adsorption data points were collected by performing the Cd(ii) adsorption measurements on MOF-DSH MOF. The adsorption capacity (*q*_e_) was measured as a function of Cd(ii) initial concentration, pH of the medium and contact time and used to train, validate and test the developed ANN model. A total of 60 data points was collected to construct the input dataset. ANN-based modeling is a multivariate calibration methodology. Hence, ANN-based modeling is intended to find the regression relationship between two matrices, *A* and *B*, related by the following relationship:

2 *B* = *f*(*A*) + *ε*

Matrix *A* is an *m* × *n* matrix, which represents the adsorption input dataset. The input dataset was constructed from ‘*m*’ no. of different samples with ‘*n*’ no. of different conditions (initial concentration, medium pH and time; *i.e.*, *n* = 3). On the contrary, matrix *B* is an *m* × *p* matrix, which consists of ‘*m*’ no. of samples with ‘*p*’ no. of different analytes (here it is the adsorption capacity) to be analyzed. The activation function, designated as *f*, was employed to introduce the non-linearity into the regression and *ε* is known as error function. The ANN-based model could reveal the hidden relationship between the input and output data given that it is a multi-input and multi-output regression model, respectively. In the presently developed methodology, the ANN model was trained, validated and tested with a calibration dataset that consists of 60 adsorption points. The standard error of prediction (SEP) parameter was used to assess the predictability of the trained model and defined as follows:3
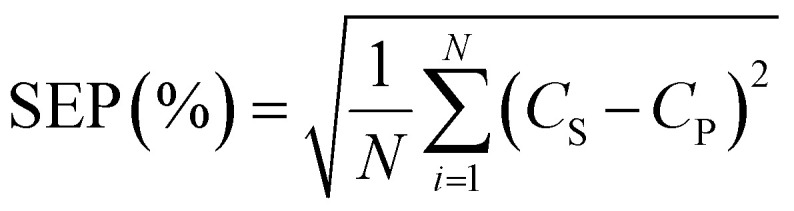
where *N* represents the number of samples used for validation; *C*_S_ denotes the standard Cd adsorption and *C*_P_ is the predicted Cd adsorption. The least SEP valued configuration was selected as the optimum number of nodes along with hidden layers. In the hidden layers, the sigmoid function was employed as an activation function. A linear activation function was used as a transfer function in the output layer. A feed forward network was built to model a non-linear regression problem. The best possible architectural configurations (*e.g.*, number of hidden layers, number of nodes, number of iterations, and activation function) were finalized by means of repetitive iteration and the best possible configurations were used for validation.

The presently developed ANN model was implemented utilizing the Python 3.9.7 version. Data analysis tools such as Pandas were employed for handling data.^33^ To handle big data, the open-source Python data analysis library is a very useful tool. The Python-based open-source package called NumPy was employed for various scientific computing (*e.g.* handling multi-dimensional arrays, processing various arrays, and various mathematical operations).^34^ For implementing the basic artificial neural network model, open-source machine learning frameworks such as Keras, TensorFlow, and scikit-learn were employed.^35–37^

## Results and discussion

**Fig. 1:**
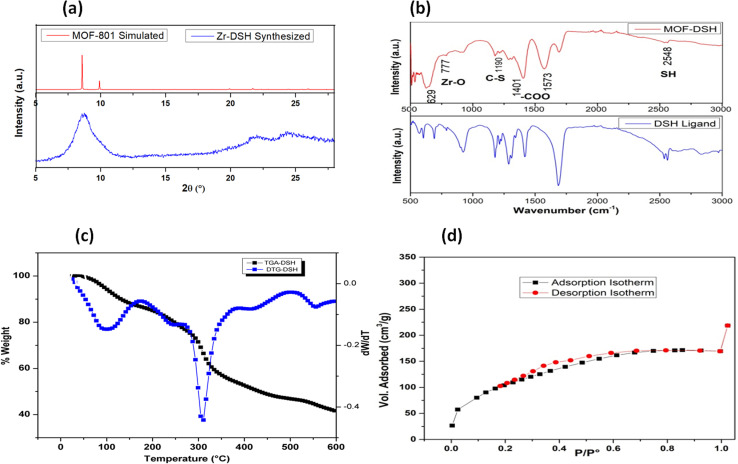
(a) PXRD pattern of MOF-DSH and simulated pattern of MOF-801 (Zr-succinate); (b) FT-IR spectra of MOF-DSH and DSH (linker); (c) thermogravimetric analysis; (d) N_2_-adsorption–desorption isotherms.

Although the XRD peaks observed were broad, indicating the semi-crystalline nature of the synthesized MOF, the observed XRD patterns were in good agreement with the simulated pattern of MOF-801, which indicated the formation of MOF with an fcu topology. This can be seen in Fig. 1(a). The broad XRD pattern might be due to the nano nature of MOF. Additionally, the presence of aliphatic linkers could also lead to broadening of the diffraction lines, as observed in the literature.^38^ The low-angle, broad diffraction peak observed could easily be deconvoluted into 2 peaks, which are characteristic for a hexa-cluster type of structure.^39^ The other peaks are in agreement with the reported data.^30^

The FT-IR spectrum of Zr-DSH MOF was recorded for ascertaining the incorporation of the thiol functionality in the MOF. For comparison, the FT-IR spectrum of the free of ligand was also recorded. Both spectra are presented in Fig. 1(b). The intense carbonyl peak (1690 cm^−1^) in the bare ligand split into symmetric (1401 cm^−1^) and asymmetric (1573 cm^−1^) stretching vibrations in the case of Zr-DSH. The presence of both these in- and out-of phase stretching modes in the synthesized MOF indicated the successful linkage of the thiol-bearing ligand functionality with a metal ion.^40,41^ The broad band corresponding to 2500–2600 cm^−1^ is related to the free –SH (2548 cm^−1^), further indicating the incorporation of the thiol group in the MOF structure. A similar spectral feature was also observed for the free ligand, which is located at approximately the same wavenumber in the case of both materials. This ensured that the thiol group was freely available for Cd(ii) capture.

To assess the thermal stability of the formed product, TGA was carried out, as depicted in Fig. 1(c). The first derivative of this data (DTG) can also be seen in Fig. 1(c). According to this figure, two main weight loss steps can be seen in the temperature range of 25–500 °C. In general, aliphatic linker-based MOFs are thermally less stable compared to MOFs built from aromatic linkers.^42^ The first weight loss at ∼85–110 °C corresponds to the loss of ethanol/water from the surface of MOF. The small hump at 220 °C may be due to the presence of water residing inside the pores, which was eliminated at a higher temperature compared to water on the surface. The second main weight loss observed at around 290–310 °C corresponds to the disintegration of the framework and removal of carbonaceous materials from MOF, which gradually decomposed to ZrO_2_ based residues with a further increase in the temperature. This study showed that the MOF was stable up to ∼300 °C.

The morphology of MOF-DSH was determined using scanning electron microscopy (SEM), which revealed the presence of small particles in a highly agglomerated state. Hence, it was difficult to determine the exact size of the particles. Their morphology was almost the same as the earlier reported literature.^30^ The SEM image is shown in ESI Fig. 3.† Further, energy dispersive spectroscopy (EDS) was carried out to confirm the presence of sulphur in the synthesized framework. ESI Fig. S4† shows the EDS spectrum and clearly shows the presence of sulphur in MOF, which confirms the availability of SH groups for the chelation of Cd(ii) metal ions. Further, to magnify the particles, FE-SEM images were captured, which showed the agglomeration of the particles, as shown in ESI Fig. S5.†

The surface area and pore volume play a crucial role in the adsorption of metal ions and the surface sites are usually correlated with the specific surface area of an adsorbent. Thus, the low-temperature N_2_-based adsorption desorption isotherms were recorded, as shown in Fig. 1(d), to calculate the specific surface area of MOF-DSH. A type-1 BET isotherm was obtained, which is characteristic of microporous materials. However, its specific surface area (∼290 m^2^ g^−1^) and pore volume (∼0.27 cm^3^ g^−1^) were significantly lower compared to that of a typical MOF bound with aromatic linkers. The observed results were comparable to the reported specific surface area and pore volume of MOFs containing similar linkers containing –SH groups. This may be due to the flexible aliphatic linker used in the present study, which is susceptible to free rotation, and as a result could occupy the pores of the synthesized framework.

### Chemical stability studies

Before performing the adsorption studies, the stability of MOF was evaluated under different pH. For this, MOF-DSH was immersed in the respective solutions for 24 h and its XRD pattern was recorded post-immersion. No considerable change in the peak position or intensity was observed in the XRD pattern under these conditions, as shown in ESI Fig. S6.† These studies ensured that the synthesized MOF is stable under the simulated aqueous conditions for the adsorption of Cd ions.

### Batch adsorption of Cd(ii)

#### pH dependence on adsorption capacity

**Fig. 2:**
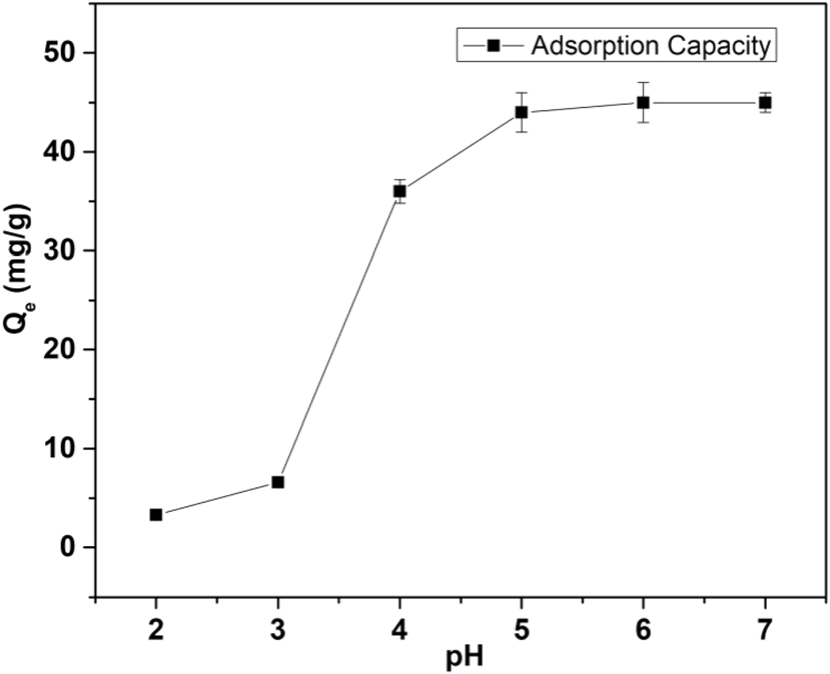
Variation in Cd(ii) adsorption capacity of MOF-DSH with change in pH. (*C*_o_ = 50 mg L^−1^; equilibration time = 5 h; pH = 2–7; *T* = 25 °C; *m*/*V* = 0.3).

The pH of a solution expressively effects the exclusion of cadmium (Cd) from a solution using a metal organic framework (MOF) given that not only it highly influences the speciation of a metal ion but also the surface charge of the adsorbent is governed by the pH conditions of the solution. Thus, the combination of these two factors eventually drives the removal of metal ions from aqueous solution. The concentration of Cd ions in solution and the affinity of Cd ions to certain functionalities present in the MOF structure also significantly influence the optimal pH at which the maximum adsorption capacity can be achieved. Generally, an increased adsorption of Cd ions could be seen at higher pH, which is ascribed to the electrostatic interactions between the positively charged metal ions and the negatively charged MOF surface at higher pH. Cd(ii) is usually known to be present as bare Cd(ii) in the pH range of 1 to 8, which further gets hydrolyzed to form hydroxide-based complexes above pH 8, as reported in the literature.^43^ In the present study, the pH was varied from 2 to 7 to ensure that only Cd(ii) existed in the solution. The initial concentration of ∼50 mg L^−1^ and equilibration time of ∼5 h were used to carry out the pH-based experiments. Fig. 2 shows the variation in adsorption capacity with a change in pH and the corresponding values are presented in ESI Table S1.† At lower pH (2–3), two factors play a decisive role in the adsorption characteristics of Cd(ii) on the surface of MOF. The first is the presence of more H^+^ in the solution to compete with Cd(ii) ions. Secondly, the adsorbent surface would be positively charged under acidic conditions.^44^ At lower pH (∼2), due to the higher concentration of H^+^ ions in solution, a large amount of H^+^ ions accumulate around the MOF surface, leading to positive charge accumulation on the surface of the adsorbent. The presence of H^+^ ions near the surface causes protonation of the –SH moieties present in the MOF as follows:MOF-DSH + H^+^ → MOF-DSH_2_^+^

These positively charged surface experience electrostatic repulsion from the positively charged Cd(ii) ions. Overall, there will be competition between the H^+^ and Cd(ii) ions, resulting in the very low adsorption of Cd(ii) ions at a lower pH. With an increase in pH, the concentration of H_3_O^+^ diminishes and so does its attraction towards the MOF. This increased pH results in deprotonation of the –SH functionality, leading to increasing electrostatic attraction towards Cd(ii) ions, hence increasing the adsorption capacity.

The adsorption results observed in our case are in accordance with the above-mentioned cited hypothesis. Up to pH 3, the adsorption of Cd(ii) observed was very low. Beyond pH 3, we observed a higher adsorption capacity. The enhanced Cd adsorption in the intermediate pH range and its decreased adsorption trend under acidic conditions is governed by the adsorption mechanism involving deprotonation of the thiol, which is further explained in the adsorption mechanism section. Beyond pH 5, the adsorption capacity was independent of pH, which suggests the saturation of adsorption capacity under the experimental conditions. Thus, to avoid hydrolysis and precipitation, further experiments were carried out at pH 6.

#### Adsorption kinetics

**Fig. 3:**
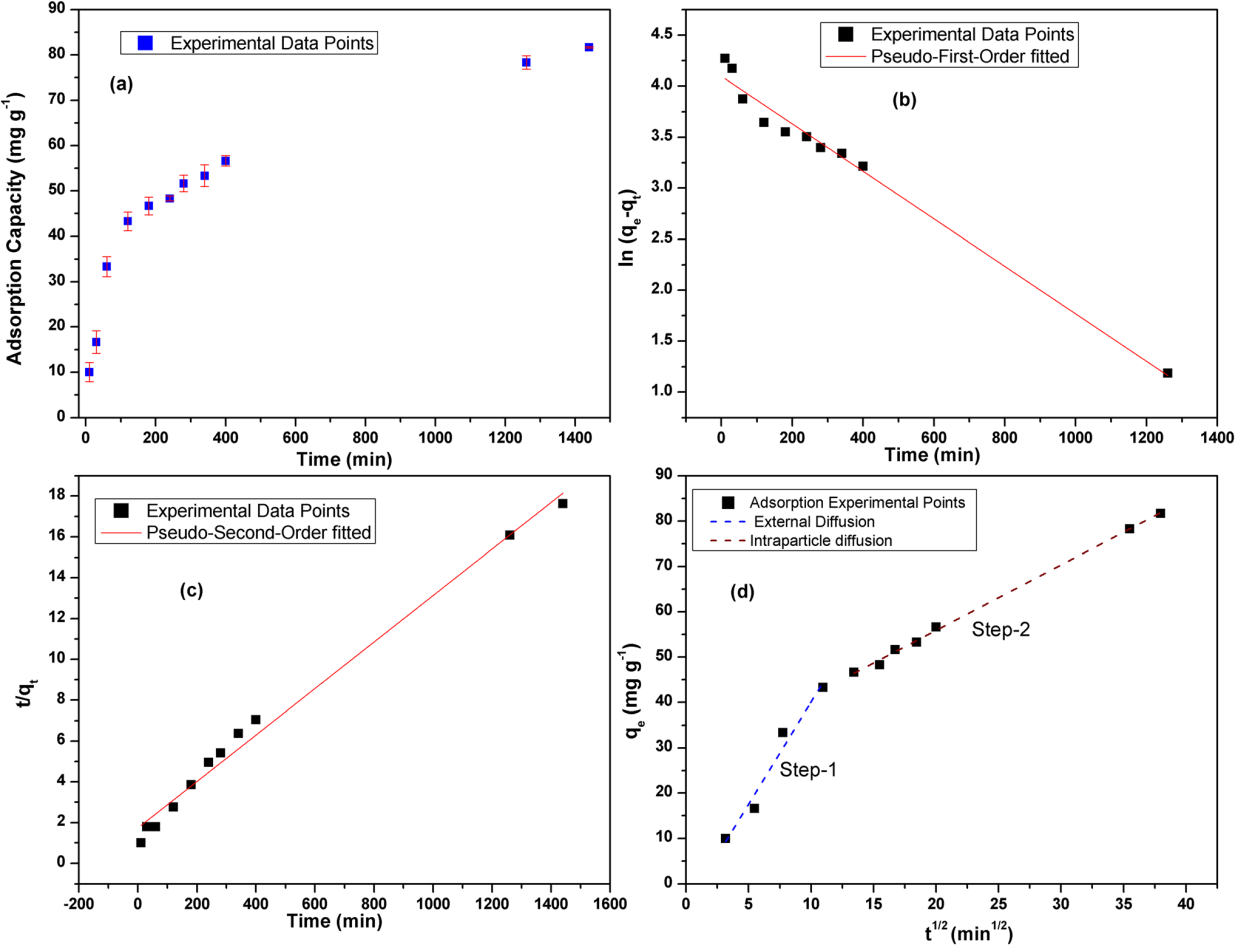
(a) Variation in Cd(ii) adsorption capacity with change in contact time; experimental points fitted using (b) pseudo-first-order; (c) pseudo-second-order; (d) intraparticle diffusion model. (*C*_o_ = 50 mg L; equilibration time = 10 min–24 h; pH = 6; *T* = 25 °C; *m*/*V* = 0.3).

Information about the kinetics of the adsorption of metal ions on MOF is a key parameter, which determines the swiftness by which the metal ions of concern can be eliminated efficiently from solution. This study is essential for designing MOFs with capacity for the rapid removal of water contaminants such as heavy metals for water purification. The role of the surface morphology of an MOF such as type, size and tunability of its pores and anchoring of various types of functionality for adsorption of metal ions of interest with increased selectivity are some of the parameters directly influencing the binding affinity and interaction strength with metal ions, impacting the adsorption rate. The fundamental criterion for an adsorbent to be utilized on the industrial scale is highly dependent on the contact time required for the equilibration between the adsorbate and adsorbent.^45^ Thus, an in-depth analysis of the contact time *vs.* adsorption capacity was evaluated at different times, as represented in Fig. 3(a), and the corresponding data is represented in ESI Table S2.† The initial concentration of Cd(ii) was fixed at 50 mg L^−1^. As observed in Fig. 3(a), during the initial 2 h, a fast increase in adsorption capacity was observed, which further became sluggish as time progressed. Nevertheless, 24 h was fixed as the equilibration time of contact to have a good contact window. The fast rate of adsorption in the initial time is ascribed to the presence of vacant sites on the adsorbent, which are filled up with time, and thus reaches saturation. Different models, namely the pseudo-first-order (PFO), pseudo-second-order (PSO) and intraparticle diffusion models, were applied to the obtained experimental data and the corresponding equations are given as (4)–(6), respectively.

4 ln *q*_e_ − ln(*q*_e_ − *q*_*t*_) = *k*_1_*t*

5
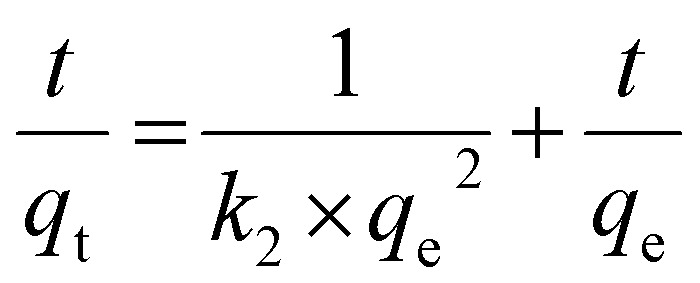

6 *q*_*t*_ = *k*_d_*t*^1/2^ + *C*

where *q*_e_ and *q*_*t*_ represent the equilibrium adsorption capacity and adsorption capacity at time ‘*t*’, respectively, in mg g^−1^. *k*_1_, *k*_2_ and *k*_d_ represent the pseudo-first-order rate constant (min^−1^), pseudo-second-order rate constant (g mg^−1^ min^−1^) and intraparticle diffusion constant (mg g^−1^ s^−1/2^), respectively. *C* represents a constant related to the Weber–Morris resistance. The experimental data fitted to the PFO and PSO models are represented as Fig. 3(b) and (c), respectively. The higher correlation factor (*R*^2^ = 0.99) obtained for PSO confirms the better suitability of this model compared to the former. This implies that the adsorption of Cd(ii) involves chemical interactions between the adsorbate and adsorbent. In addition to the goodness of fit parameter, the other parameters obtained from all the models are presented in Table 1. The closeness of *q*_e_ obtained experimentally to the PSO model further confirms the chemisorption/PSO model.

**Table 1:** Parameters obtained through Cd(ii) adsorption on MOF using pseudo-first and pseudo-second order kinetics and intraparticle diffusion models

Pseudo-first-order model			Intraparticle diffusion				Pseudo-second-order model		
*q*_e_ (mg g^−1^)	*k*_1_ (min^−1^)	*R* ^2^	*k*_1_ (mg g^−1^ s^−1/2^)	*R*_1_^2^	*k*_2_ (mg g^−1^ s^−1/2^)	*R*_2_^2^	*q*_e_ (mg g^−1^)	*k*_2_ (g mg^−1^ min^−1^)	*R* ^2^
60.1	0.0023	0.98	4.5	0.95	1.44	0.99	87.7	7.5 × 10^−5^	0.99

The intraparticle diffusion model should be considered whenever a long equilibration time is observed.^46^ Thus, to gain a deeper understanding of the adsorption kinetics, the above-mentioned model was applied. The presence of multi-linearity in Fig. 3(d) represents the involvement of 2 or more processes in the adsorption mechanism and none of the linear portions pass through the origin, which implies that intraparticular diffusion is not the sole mechanism of adsorption. The first linear region represents faster diffusion of Cd(ii) ions to the external surface of MOF-DSH. The second region implies slower intraparticle diffusion with a smaller slope (rate constant) compared to the former step, as shown in Table 1, which is due to the slowing down of intraparticle diffusion with time.^47^ The kinetics of the second step is usually dependent on different aspects of the system such as particle size and adsorbent concentration.^48^

#### Adsorption isotherm

**Fig. 4:**
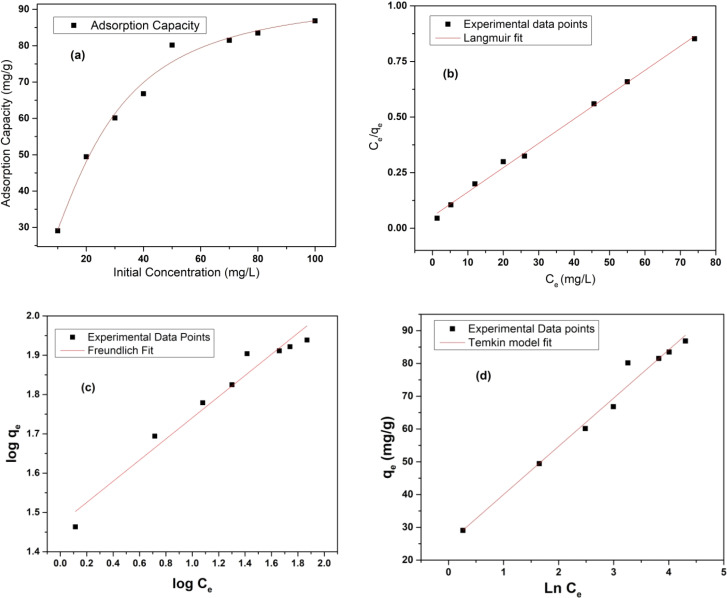
(a) Variation in Cd(ii) adsorption capacity with change in initial concentration of Cd(ii); experimental points fitted using (b) Langmuir model; (c) Freundlich model; and (d) Temkin model. (*C*_o_ = 10–100 mg L; equilibration time = 24 h; pH = 6; *T* = 25 °C; *m*/*V* = 0.3.).

The adsorption isotherm study is vital given that it portrays a thorough picture about the amount of metal ions that can be adsorbed onto MOF at a particular metal ion concentration by which one can forecast the ideal conditions for metal removal for environmental remediation. By analyzing the isotherm curve, one can determine the maximum adsorption capacity of the adsorbent and the underlying chemical interactions involved between the adsorbate and the adsorbent such as monolayer and multi-layer interactions. Thus, to estimate the maximum adsorption capacity of MOF-DSH, adsorption isotherm studies were carried out. Here, the correlation between the equilibrium capacity of the MOF and concentration was evaluated. The initial concentration of Cd(ii) was varied from 10 mg L^−1^ to 100 mg L^−1^ and the equilibrium adsorption capacity was evaluated, as shown in Fig. 4(a). As observed in this figure, the adsorption capacity was lower at a lower initial concentration of Cd(ii), which found to increase with an increase in the Cd(ii) concentration and reached a plateau beyond ∼70 mg L^−1^. This could be due to the saturation of the adsorption sites on MOF-DSH. The experimental data obtained was fitted using different models, namely the Langmuir, Freundlich and Temkin isotherm models.^49^ The linear equations for the above-mentioned models are shown as eqn (7)–(9), respectively.7
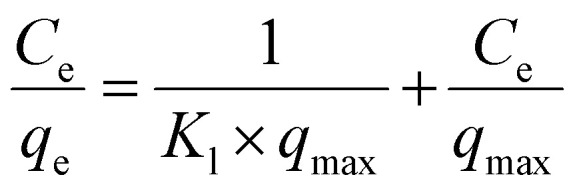
8
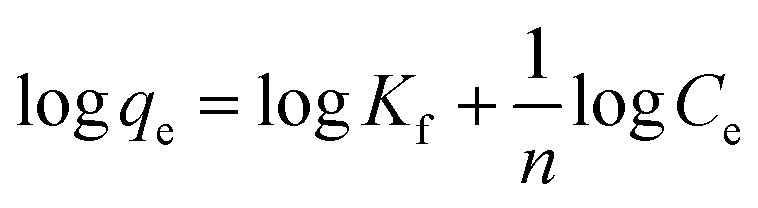
9
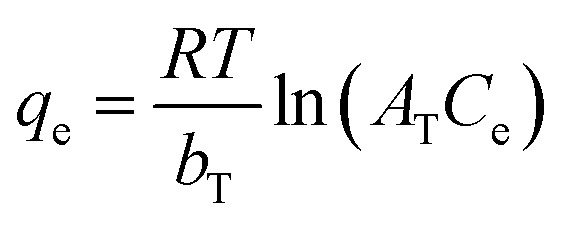
where *q*_max_ and *q*_e_ represent the maximum adsorption capacity and adsorption capacity at equilibrium in mg g^−1^, respectively. *C*_e_ represents the concentration of Cd(ii) after equilibration. *A*_T_ and *b*_T_ represent the equilibrium binding constant and Temkin isotherm constant, respectively. *T* and *R* are the temperature and ideal gas constants, respectively. The Langmuir and Freundlich models assume that monolayer homogenous and multilayer heterogeneous adsorption active sites, respectively, are responsible for the adsorption of metal ions. The Temkin isotherm assumes the interaction between the adsorbate and adsorbents, where the Gibbs energy is dependent on the surface coverage. The higher the adsorption on the surface, the lower the chances of further adsorption.^50^ Further details of the above-mentioned 3 models can be obtained elsewhere.^51–53^

The experimental data fitted using the Langmuir, Freundlich and Temkin isotherm models are shown in Fig. 4(b)–(d), respectively, and the corresponding parameters obtained are presented in Table 2. The maximum adsorption capacity, *q*_max_ (at pH 6), observed was 91.5 mg g^−1^. An obviously better fit and correlation coefficient were observed in the case of the Langmuir model, which suggests the involvement of monolayer active sites present on a homogenous surface. Further, a parameter known as *R*_l_, which is defined by eqn (10) to predict the favorability of adsorption, was evaluated for different initial concentrations of Cd(ii) and tabulated in ESI Table S3 and shown in ESI Fig. S7.†*R*_l_ represents the evolution of the reaction and is a unit less equilibrium factor calculated from the Langmuir binding constant, *K*_l_ (L mg^−1^), and initial concentration of Cd(ii), *C*_o_.10
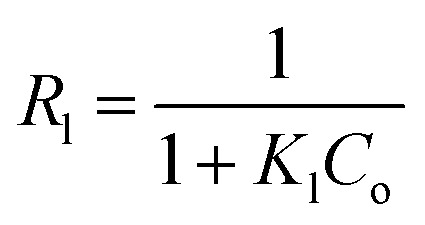


**Table 2:** Parameters obtained by Cd(ii) adsorption on MOF-DSH using Langmuir, Freundlich, and Temkin isotherms

Langmuir isotherm			Freundlich isotherm			Temkin isotherm		
*q*_max_ (mg g^−1^)	*K*_l_ (L mg^−1^)	*R* ^2^	*n*	*K*_f_ (L mg^−1^)^1/*n*^ (mg g^−1^)	*R* ^2^	*b*_T_ (kJ mol^−1^)	*A*_T_ (L mg^−1^)	*R* ^2^
91.5	0.205	0.996	3.71	29.62	0.955	168.4	5.56	0.974

The feasibility/equilibrium of adsorption is dependent on the magnitude of *R*_l_, which is a function of the adsorption energy. Different processes such as unfavorable (*R*_l_ > 1), favorable (0 < *R*_l_ < 1), irreversible (*R*_l_ = 0) and linear (*R*_l_ = 1) adsorption were observed.^54^ Over the whole range of initial concentrations studied, the *R*_l_ value lies between 0 and 1, which indicates the favorable adsorption of Cd(ii) on MOF-DSH. At higher concentrations, the value skewed towards 0, which indicates the higher degree of favorability at a higher concentration.^55^

#### Reusability studies

**Fig. 5:**
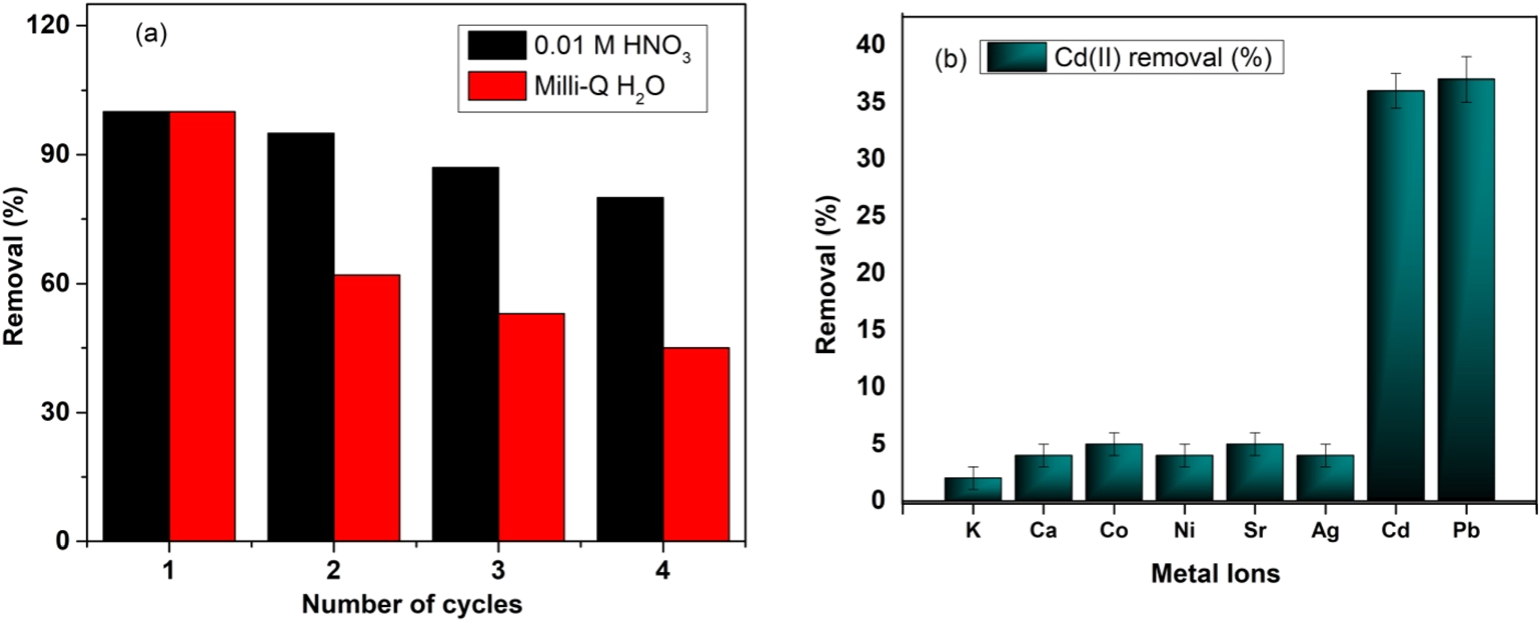
(a) Adsorption–desorption behavior of MOF-DSH for different cycles with different eluents; and (b) removal (%) of different metal ions present together in aqueous solution using MOF-DSH.

It is important to evaluate the frequency that an MOF can be reused without compromising its adsorption performance, which is essential for its cost-effective application and sustainable usage. This is normally carried out by multiple adsorption–desorption processes, which are normally correlated with strength of the bond between metal nodes and organic linker. This ultimately contributes to the stability and durability of the MOF for a particular adsorption experiment. The reusability of MOF-DSH was analyzed through adsorption desorption cycles and the adsorption removal in the 1st cycle was considered as 100% removal. Different eluents such as dilute HNO_3_, Na_2_CO_3_ and Milli-Q were used and the best results were obtained using 0.01 M HNO_3_ and the comparative analysis with the Milli-Q water is shown in Fig. 5(a). Specifically, ∼80% of the adsorption sites were available when 0.01 M HNO_3_ was used as the eluent, in contrast to 45% of available sites in the case of Milli-Q water subsequent to four continuous adsorption desorption cycles. This can be attributed to the fact that the adsorption capacity is lower at a lower pH (pH = 2 for 0.01 M HNO_3_), as observed in Fig. 2, facilitating the elution in the presence of a large amount of H_3_O^+^. In contrast, at the pH of Milli-Q water (pH ∼6–7), the adsorption capacity towards Cd(ii) was high, and thus very difficult to elute from the surface of MOF-DSH.

#### Interference from other ions

Cations, particularly with analogous chemical properties and similar charge to Cd(ii) ions, can compete for the surface of MOF when they co-exist in solution. Thus, a study of the removal of Cd(ii) ions in the presence of many co-existing metal ions was undertaken by preparing a stock solution containing equimolar amounts of commonly found ions such as K^+^, Ca^2+^, Co^2+^, Ni^2+^, Sr^2+^, Ag^+^, Cd^2+^ and Pb^2+^. The removal % of different metal ions is shown in Fig. 5(b) and the corresponding values are presented in ESI Table S4.† Given that Ca^2+^ has a similar charge and smaller hydrated radius compared to the Cd^2+^ ion, it was expected to have greater affinity for the adsorbent surface. Except for Pb^2+^, all the other elements were adsorbed insignificantly on the surface of MOF. Given that Cd^2+^ has a larger ionic radius and more polarizable electron cloud compared to Ca^2+^, the former is softer compared to the latter according to the hard–soft acid base (HSAB) theory, and the presence of softer ligand functionality (–SH) in the MOF facilitates the preferential adsorption of Cd^2+^ compared to Ca^2+^. Although both Pb^2+^ and Cd^2+^ are considered soft acids, Pb^2+^ displayed slightly higher adsorption tendency than Cd^2+^ due to its larger size and softer nature.^44,56,57^ In future, efforts will be made to induce higher selectivity for Cd(ii) through means of ligand functionalization/composite preparation.

#### Cd coordination mechanism with MOF-DSH

To understand the coordination mechanism of Cd(ii) ions with MOF-DSH, XRD, FT-IR, pH studies and XPS were carried out pre- and post-adsorption.

#### XRD studies

The stability of MOF post-Cd adsorption was evaluated by recording its XRD pattern post-Cd adsorption and through XRD measurement and it was compared with that of the as-synthesized MOF. The similar XRD peaks before and after Cd(ii) adsorption in MOF-DSH confirm the stability of MOF under the experimental conditions, as shown in ESI Fig. S8.†

#### FT-IR studies and pH change post adsorption

**Fig. 6:**
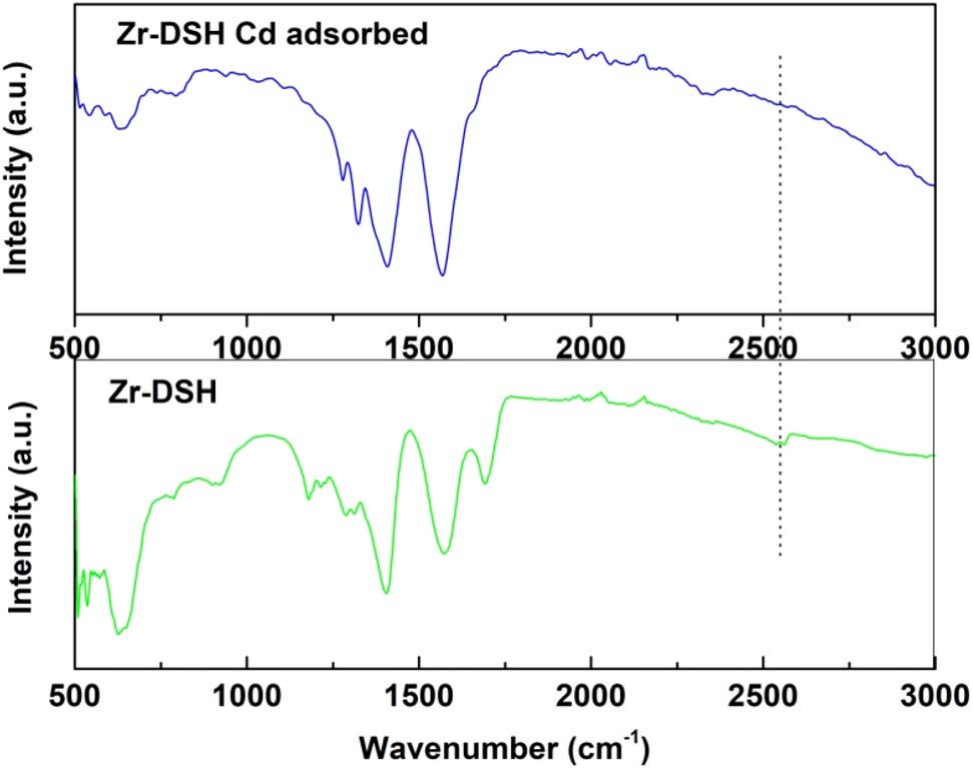
FT-IR spectra of MOF-DSH before and after Cd(ii) adsorption.

To understand the mechanism of adsorption, the FT-IR spectrum post-Cd adsorption was recorded and compared with that of the bare MOF, as shown in Fig. 6. Besides the thiol vibrational peak, almost all the other peaks were present after Cd(ii) adsorption. The peak at ∼2548 cm^−1^ for MOF-DSH disappeared after adsorption, which indicates the involvement of the –SH moiety for Cd adsorption.^58^ The dotted line in Fig. 6 is shown for reference and clearly elucidates the absence of an –SH peak. Further, we hypothesized the formation of the S–Cd bond through the ion exchange mechanism. To confirm this, pH studies prior and post-Cd adsorption were performed and a decrease in pH from 6 (before adsorption) to 3.4 was observed post-Cd(ii) adsorption, which confirms that ion exchange is the mechanism prevailing.^59^ The same could not be observed in the FT-IR spectra given that the Cd–S vibrational frequency is expected at ∼500–650 cm^−1^ and could not be distinguished because of the higher background in the far-IR region.^60^

#### XPS studies

**Fig. 7:**
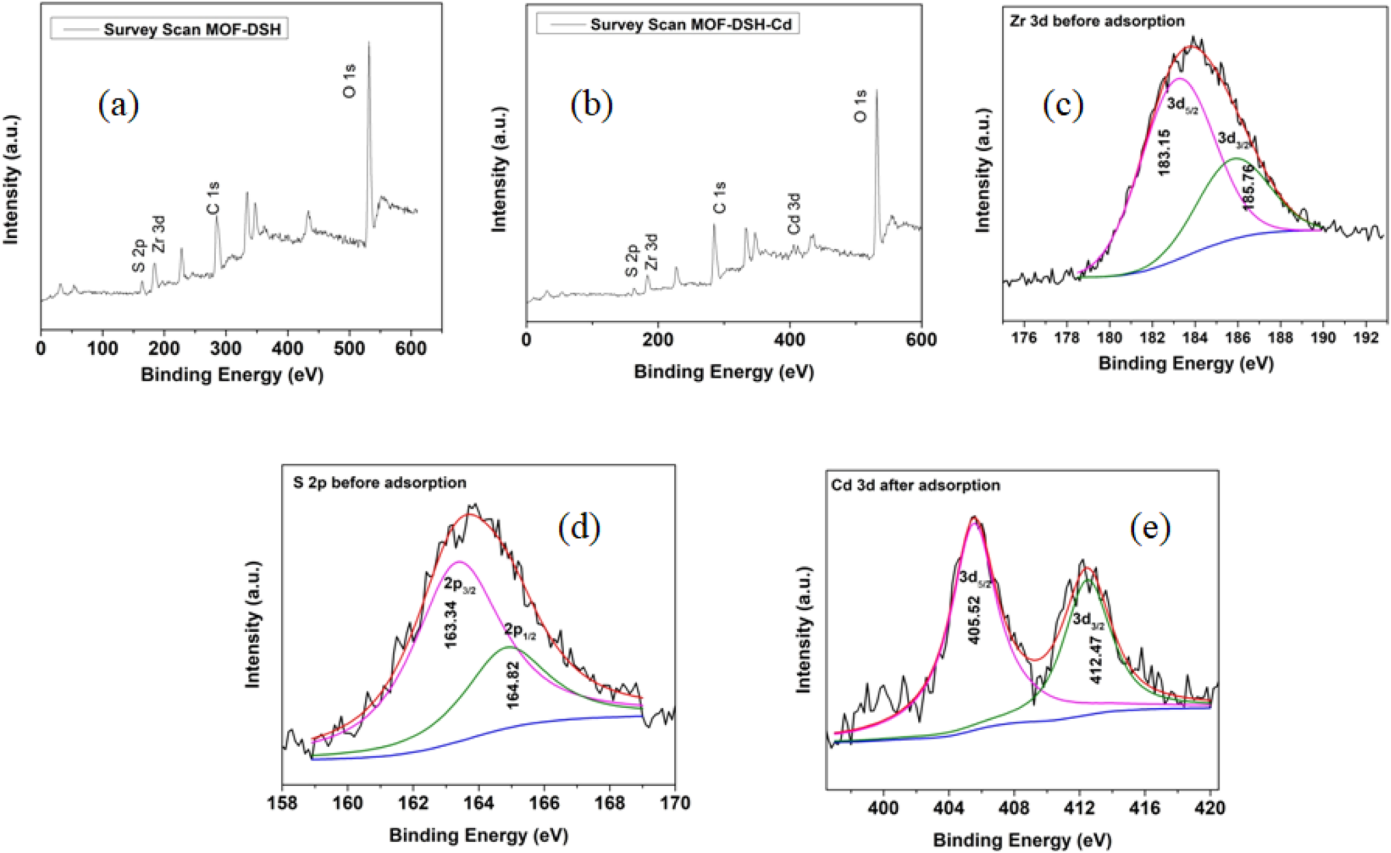
XPS (a) survey scan of MOF-DSH before adsorption; (b) survey scan of MOF-DSH after Cd(ii) adsorption; (c) Zr 3d spectrum before adsorption; (d) S 2p spectrum before adsorption; and (e) Cd 3d spectrum after adsorption.

Fig. 7(a) and (b) present the survey scan XPS spectra of MOF-DSH and MOF-DSH Cd-adsorbed, respectively. The presence of all the elements such as S, Zr, C and O could be established by observing the XPS peak at their respective positions, which also corroborates our characterization results, as discussed earlier. Post-Cd(ii) adsorption, a few extra peaks were observed with the other elements present at almost the same position, which confirms the stability of the framework. In addition, naked-eye observation also confirmed the decrease in the intensity of the S 2p peak, which suggests the involvement of S in the adsorption of Cd(ii), hence governing the adsorption mechanism.

The Zr 3d spectra before and after adsorption are shown in Fig. 7(c) and ESI S9(a),† respectively. The Zr 3d peak was also deconvoluted into 2 peaks, corresponding to 3d_5/2_ and 3d_3/2_ in the ratio of 3 : 2, respectively. The peak positions of both peaks are in agreement with the reported literature.^61,62^ Post-Cd(ii) adsorption, an insignificant shift in the binding energy was observed, as shown in ESI Fig. S9(a).† This also confirms the negligible role of Zr–O/Zr clusters in Cd(ii) adsorption, which is consistent with the HSAB principle, where a relatively softer acid Cd prefers a softer Lewis base such as S rather than a stronger Lewis base O.

To further clarify the adsorption mechanism, the S 2p peak was magnified, as shown in Fig. 7(d). The S 2p peak could be deconvoluted into 2 peaks, corresponding to 2p_3/2_ and 2p_1/2_ at 163.34 and 164.82 eV, respectively, and were in the ratio of 2 : 1 according to the L–S coupling rules.^63^ After the adsorption of Cd(ii), the intensity of the S 2p peak was diminished and the deconvoluted S 2p spectrum post-Cd adsorption is presented in ESI Fig. S9(b).† Two different factors may influence the shape and position of the peaks observed. Firstly, the shape of the peaks obtained was not symmetric, as can be seen through the raw data as well. This is attributed to the possibility of the presence of a mixture of S–Cd and S–H (in a smaller amount) together. Thus, this gives rise to a non-Gaussian distribution of peaks. Further, the peak positions were also red-shifted, which indicates the formation of the S–Cd bond.^64^ The lower electronegativity of Cd (in S–Cd) compared to hydrogen (in S–H) would lead to a higher electron density at S compared to the latter, and hence a lower energy is needed to excite the electron from the 2p orbital of S. This is consistent with the FT-IR results.

Lastly the successful loading of Cd(ii) on MOF was confirmed through the observation of the Cd 3d_5/2_ and 3d_3/2_ peaks at 405.52 and 412.47 eV, respectively, which are consistent with the observations reported by different researchers.^65–67^ The 3d XPS spectrum of Cd is represented in Fig. 7(e).

**Fig. 8:**
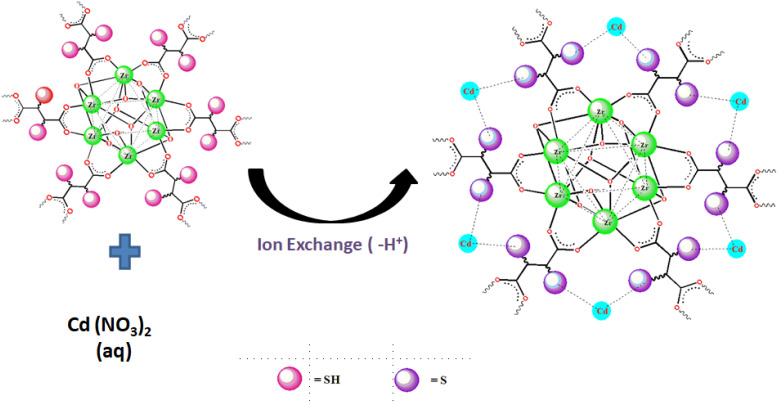
Pictorial representation of proposed adsorption mechanism of Cd(ii) on MOF-DSH.

According to both the XPS and FT-IR results, we proposed the ion exchange mechanism mode as the predominant mode of interaction of Cd(ii) with MOF-DSH, which is represented pictorially in Fig. 8. The decrease in pH post-Cd(ii) adsorption due to the release of H^+^ ions also corroborates the XPS and FT-IR results.

#### Modeling the Cd adsorption behavior of thiol-based MOF-DSH MOF using ANN

The functionality of a neural network follows the human brain. It consists of multiple nodes given that the brain consists of multiple biological neurons. The nodes accept the input data and perform the optimum response after performing iterations. In this way, the network is trained to produce a response for an unknown input dataset. The input data set was comprised of a matrix (*X*_60×3_) in which the row and column indicate the different measurements and the no. of controlling parameters (initial concentration, pH and contact time), respectively. An unbiased model was built by slicing the input data set into training, validation, and test data sets in a ratio of 60 : 20 : 20. Hence, the input dataset was sliced into training (*X*^train^_36×3_), validating (*X*^validation^_12×3_) and testing data sets (*X*^test^_12×3_).

To train and validate the ANN model, the (*X*^train^_36×3_) and (*X*^validation^_12×3_) matrices were used. The performance of the model was tested on the (*X*^test^_12×3_) test dataset. The optimization process involved (1) optimization of the no. of hidden layers along with the no. of hidden nodes, (2) activation function and (3) no. of iterations (*i.e.*, no of epochs) for finalizing the ANN architecture. A manual optimization procedure was followed to optimize the ANN architecture. The manual optimization process involved the optimization of the ANN configuration and hyperparameters through the trial and error method. The detailed methodology can be found elsewhere.^68^

We considered four ANN configurations with 25, 50, 75, and 100 neurons and the hidden layers were varied from one to four. Hence, a total 16 different configurations was examined for optimizing the ANN architecture. The details of the different ANN configurations are tabulated in ESI Table S5.† Three input nodes for each configuration were considered in the manual optimization process as the number of different experimental conditions for adsorption study and the number of output node is 1 (for the corresponding Cd adsorption capacity by the thiol-based MOF-DSH for the considered condition). Various numbers of hidden layers along with hidden nodes exist between the input and output layers. Optimization of the ANN architecture was performed by varying the number of hidden layers. The sigmoid function was considered as the activation function and the SEP parameter for each configuration was calculated. ESI Fig. S10(a)† presents the functional dependency of SEP on the neuron number. The SEP for each configuration was determined by averaging 10 different ANN analyses with identical analytical conditions. It can be concluded from the plots that the minimum SEP value configuration is 75 neurons with a different number of hidden layers. It can further be confirmed that 200 neurons with 3 hidden layers exhibit the minimum SEP value configuration. Hence, for the modeling of Cd adsorption by the thiol-based MOF-DSH, the optimum ANN configuration should be 3-50-20-5-1. All the ANN analyses were carried out with epochs of 10 000. After the architectural optimization, the activation function was also optimized by keeping the minimum SEP value ANN configuration. The trial activation functions for the optimization were sigmoid, Tanh and ReLu. The mathematical expressions for the mentioned function are as follows:11
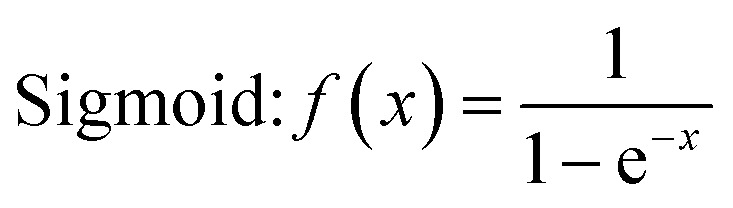
12
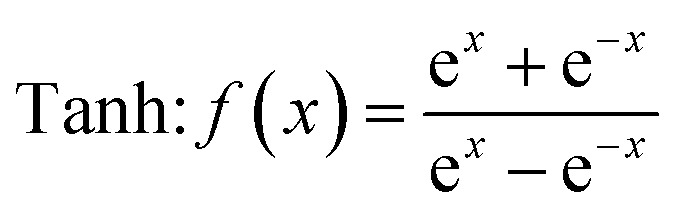

13 ReLu: *f*(*x*) = max(0,*x*)

ESI Fig. S10(b)† shows the variation in the SEP value with different activation functions. Among them, the sigmoid activation function is the best for Cd adsorption by the thiol-based MOF-DSH due to the close grouping of the SEP values. Finally, the number of iterations (epochs) was optimized. For this purpose, the optimized ANN architecture together with the activation function was fixed and the training and validation was done by varying the no. of epochs. ESI Fig. S10(c)† suggests that the critical iteration number is 3000 given that there is no significant change in SEP value after that.

**Fig. 9:**
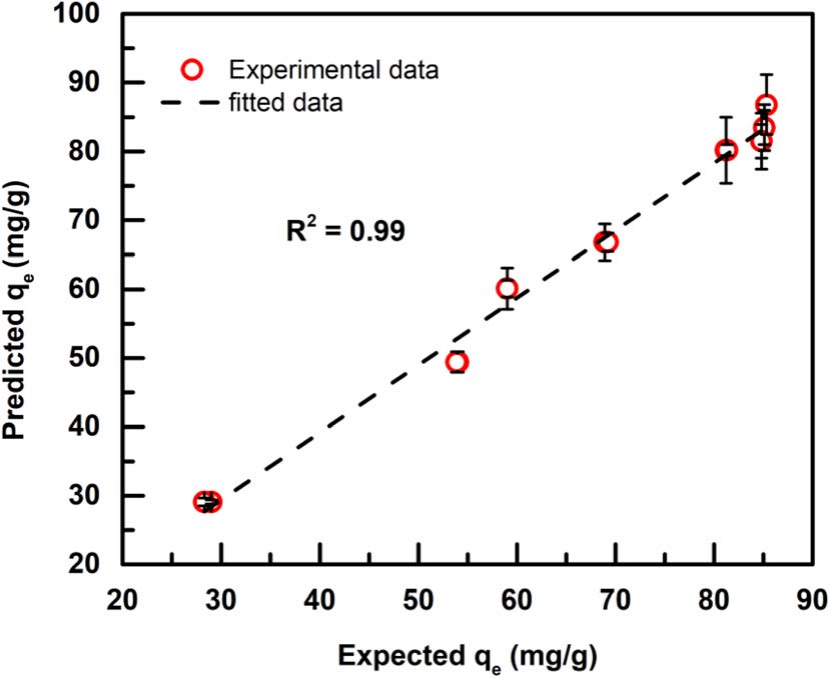
Validation plots for predicted *q*_e_.

After optimizing all the parameters (*i.e.*, ANN architecture, activation function and epochs no.), the relative error and precision for the predictability of Cd adsorption capacity by the thiol-based MOF-DSH as a function of Cd concentration, contact time and medium pH were evaluated in test dataset using the ANN model with the optimized parameters. For tracking the losses, a regression loss function named mean square error was used. To optimize the losses, the Adam optimizer, a variant of the stochastic gradient descent method, was employed. After all the parameter optimizations, the test samples were run and the average error and precision obtained using the ANN based methodology are 2.3% and 3.0%, respectively. Fig. 9 presents the validation plot of the presently developed ANN model on the test dataset. *R*^2^ ∼ 0.995 indicates the good validity of the model for predicting the parameter *q*_e_ as a function of Cd(ii) initial concentration, pH of the medium and contact time.

#### Comparison with other materials and future potentials

Table 3 shows a comparison of MOF-DSH with other potential adsorbents reported in the literature for Cd(ii) adsorption and it can be found that its adsorption capacity is comparable with that reported for other materials. However, most of the reported adsorbents have a lower capacity, thus limiting their use. Given that an aliphatic linker was used in our study, the surface area was smaller in contrast to aromatic linker-based MOFs, and thus in the future, benzene-derived ligands can be utilized, which may increase the surface energy, and eventually lead to a higher adsorption capacity. In another strategy, the preparation of composites with GO/activated carbon/silica and other porous materials may result in a higher uptake due to their synergistic effect, as reported for different materials in the literature.^69^

**Table 3:** Literature comparison of adsorption capacity of different materials towards Cd(ii)

Adsorbent	Adsorption capacity (mg g^−1^)	Mass of adsorbent/volume of Cd(ii) solution (g L^−1^), initial concentration of Cd(ii) in solution	pH, *T*	Reference
HS-mSi@MOF-5	98.02	0.05, N.A.	6, 283 K	21
Cu_3_(BTC)_2_–SO_3_H	88.7	1, 200 mg L^−1^	6, 298 K	22
Cu-MOF	9.27	10, 100 mg L^−1^	4, 303 K	70
ED-MIL-101 (Cr)	62.5	1.2, 200 mg L^−1^	6, 298 K	71
MnFe_2_O_4_@SiO_2_@VTMS	126	0.04, 150 mg L^−1^	6, 298 K	72
TMU-4	48	0.56, 100 μg L^−1^	10, 300 K	73
MIL-53(Fe)-1	714.28			74
HKUST-1-MW@H3PW12O40	32.80	2, 200 mg L^−1^	7, 303 K	75
MBC800-0.630	46.9	1, 150 mg L^−1^	6, N.A.	76
Lig800	91.3	1, 100 mg L^−1^	5.3, 298 K	77
**MOF-DSH**	**91.5**	**0.3, 100 mg L^−1^**	**6, 298 K**	**This work**

## Conclusions

A thiol-functionalized zirconium-based MOF (MOF-DSH) was synthesized *via* the conventional heating method for the adsorption of Cd(ii) from aqueous solution. Its XRD pattern showed low angle diffraction peaks characteristic of hexa-cluster and the peaks were broader owing to the aliphatic linkers present in the network. Due to the presence of aliphatic linkers, the BET surface area exhibited by the MOF was low, which was reflected in its adsorption characteristics, and also its maximum adsorption capacity of 91.5 mg g^−1^. Although this value is not poor compared with many of the reported works, we anticipate a better adsorption capacity will be achieved if we include aromatic chains with a similar thiol functionality. The pH variation studies showed that pH is one of the important parameters influencing the adsorption characteristics of Cd(ii) on MOF-DSH, and due to the speciation of Cd(ii) and surface charge characteristics of the adsorbent, the maximum adsorption was observed at pH 6. The interactive effect of process variables such as amount of adsorbent, initial concentration of Cd(ii) and its contact time with MOF-DSH was found to be highly important in evaluating the optimum equilibrium adsorption characteristics. The adsorption kinetics revealed a two-step adsorption mechanism, which explained the initial faster diffusion of Cd(ii) ions on the external surface of MOF-DSH, followed by comparatively slower intra-particle diffusion as time progressed. The coordination mechanism of Cd(ii) with MOF studied using FT-IR and XPS showed a favorable coordination at the thiol site, which is consistent with the HSAB principle, *i.e.*, soft acid–soft base coordination, and showed the feeble role of Zr–O/Zr clusters in Cd(ii) adsorption. The decrease in the pH value from 6 to 3.4 in post-Cd(ii) adsorption provided conclusive evidence to predict the interactive mechanism as the ion exchange type, which released protons. Although many of the co-existing divalent ions did not show any significant coextraction behavior, because of the higher ionic radius and more polarizability of Pb^2+^, it resulted in adsorption characteristics surpassing the adsorption capacity for Cd(ii). The elution cycles of Cd(ii) ions from MOF showed that 0.01 M HNO_3_ gave the best results as the eluting medium, removing more than 80% of adsorbed Cd(ii) ions from the surface. This enabled its effective reutilization for the sequestration of Cd(ii) from aqueous solution compared to Na_2_CO_3_ and Milli-Q water. Finally, the artificial neural network-based (ANN) methodology employed to model the adsorption capacity of Cd(ii) to predict the adsorption capacity as a function Cd concentration, time of contact and pH of the medium showed that the average error and precision obtained using the ANN-based methodology were 2.3% and 3.0%, respectively.

## Data availability

The data are available from the corresponding author upon reasonable request.

## Author contributions

(1) Nitin Gumber: conceptualization, experiment, first draft (2) Buddhadev Kanrar: artificial neural network study (3) Jaspreet Singh: XPS measurement (4) Jitendra Bahadur: EDX and FESEM (5) Rajesh V. Pai: supervision, final draft.

## Conflicts of interest

The authors declare no conflicts of interest.

## Supplementary Material

RA-015-D5RA00002E-s001
