# Supplementary material for: Unraveling the adsorption potential of Zr dithiol (MOF-DSH) through experimentation and neural network modeling
Source: RSC Adv. 2025 Apr 15;15(15):11811–25. doi: 10.1039/d5ra00002e (PMC11998089; doi:10.1039/d5ra00002e)
Supplement: RA-015-D5RA00002E-s001 [file RA-015-D5RA00002E-s001.pdf]

Supplementary Information (SI) for RSC Advances.
This journal is © The Royal Society of Chemistry 2025

Unraveling the adsorption potential of Zr dithiol (Zr-DSH) based MOF through experiment and neural network modeling

Nitin Gumber[a,d], Buddhadev Kanrar[a], Jaspreet Singh[b], Jitendra Bahadur[c,d] and Rajesh V. Pai[a,d,*]

[a]Fuel Chemistry Division, Bhabha Atomic Research Centre, Mumbai, India- 400085

[b]Technical Physics Division, Bhabha Atomic Research Centre, Mumbai, India – 400085

[c]Solid State Physics Division, Bhabha Atomic Research Centre, Mumbai, India-400085

[d]Homi Bhabha National Institute, Anushaktinagar, Mumbai, India- 400094

*Corresponding author email: rajeshvp@barc.gov.in

TXRF Measurements. The TXRF measurements were carried out using an Atominstitut, Vienna, low Z– high Z TXRF spectrometer. The instrument has Rh and Cr target X-ray tubes fitted with Pd/B_4C and Ni/C multilayers respectively. The X-ray tubes along with multi-layers can be slided and fitted in a sample chamber sequentially and used as per the experimental requirements in air and vacuum atmosphere. For the present study the Rh K_α monochromatic beam obtained from the Rh target X-ray tube was used.[1] The details of the instrumentation can be found elsewhere.[2] All the TXRF measurements were carried out at a tube voltage of 50 kV, tube current of 700 μA and with live time of 1000s. Quartz sample supports having diameter of 30 mm and thickness of 3 mm were used for sample deposition. A fixed volume of Ti having a known concentration is added as an internal standard, with respect to which the concentration of cadmium is being calculated.[3] Sample volume of 5 μL was pipette out and deposited on clean quartz sample supports in triplicate. Then they were dried on a hot plate to form a thin layer of sample. These sample specimens were placed inside the TXRF spectrometer and measured for 600s. The TXRF spectra obtained were analyzed using PyMca software.[4]

XPS Instrumentation. XPS studies were carried out under high vacuum conditions at Beam Line-14 consisting of double crystal of Si as a monochromator operational with hemispherical analyzer and an X-Ray source of energy 4.360 KeV at Synchrotron Radiation facility, RRCAT, Indore, India.

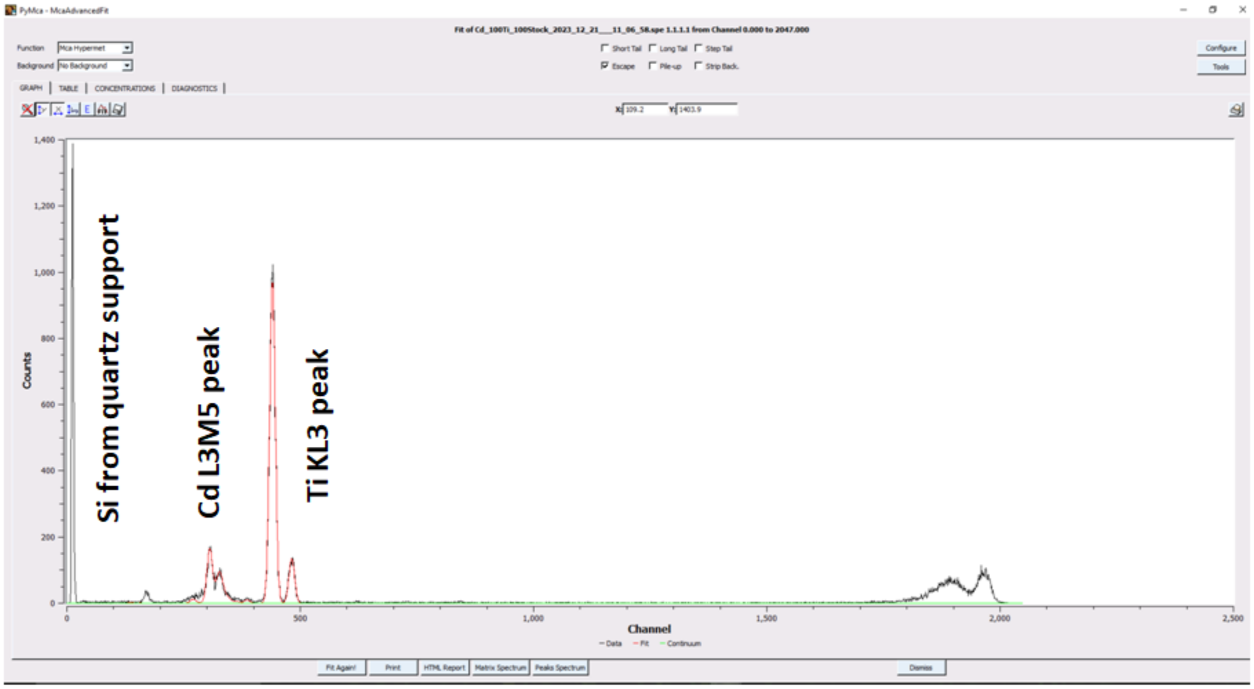

Supplementary Fig. S1: TXRF spectrum analyzed using PyMca for Cd (II) concentration in stock solution

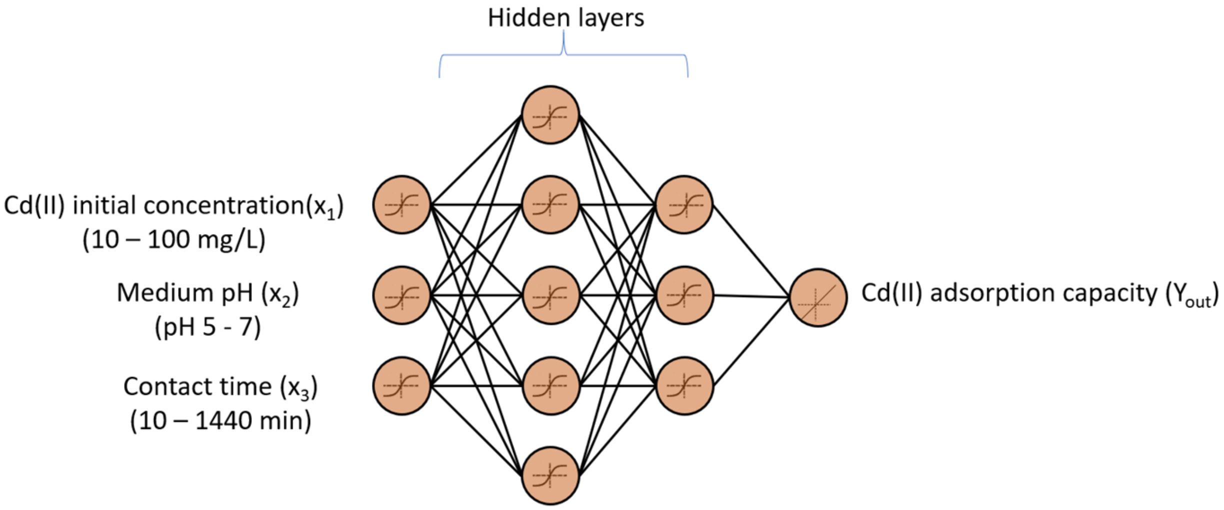

Supplementary Fig. S2: Schematic architecture of a feed forward neural network employed for the present study

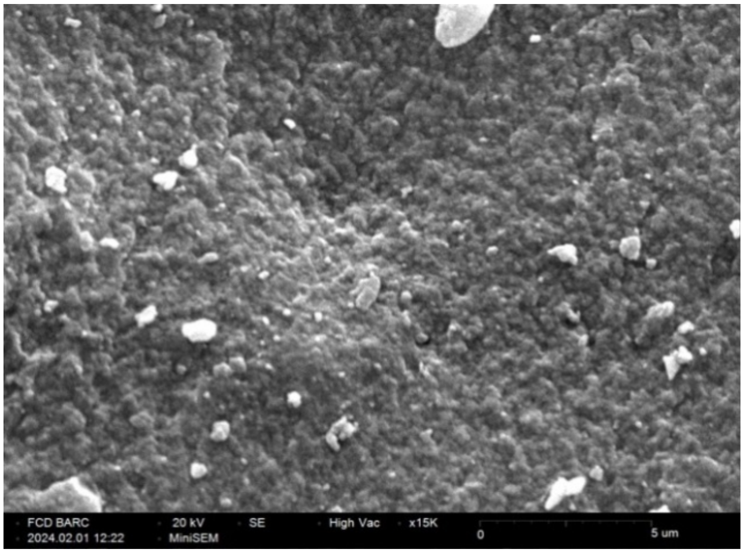

Supplementary Fig. S3: SEM image of MOF-DSH

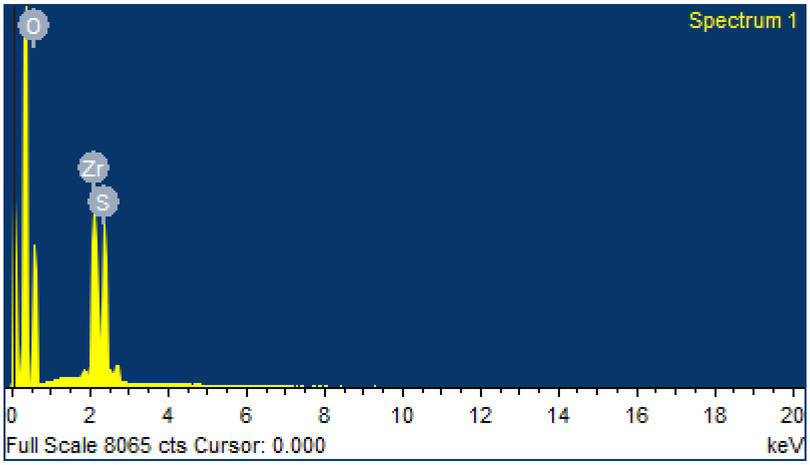

Supplementary Fig. S4: EDS spectrum of MOF-DSH showing the presence of sulfur element

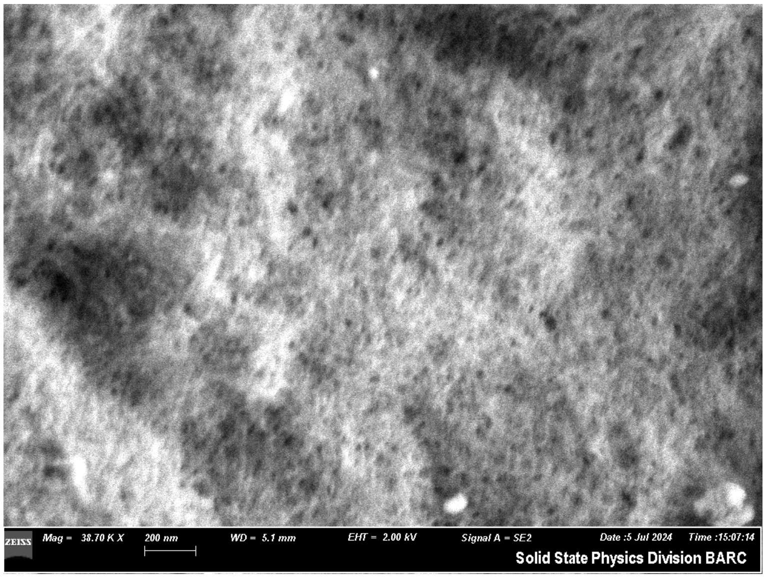

Supplementary Fig. S5: FE-SEM image of MOF-DSH

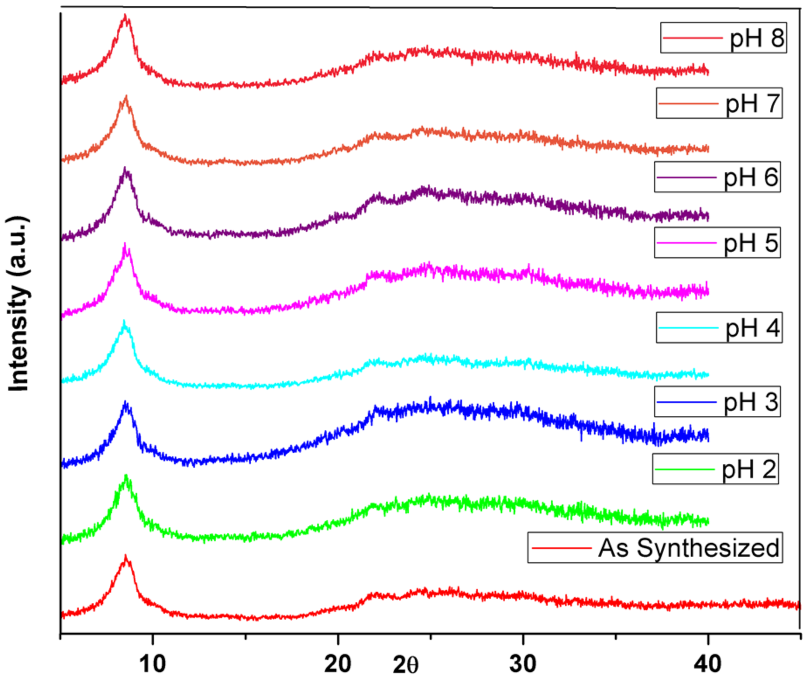

Supplementary Fig. S6: Effect of different pH on crystal structure of MOF-DSH

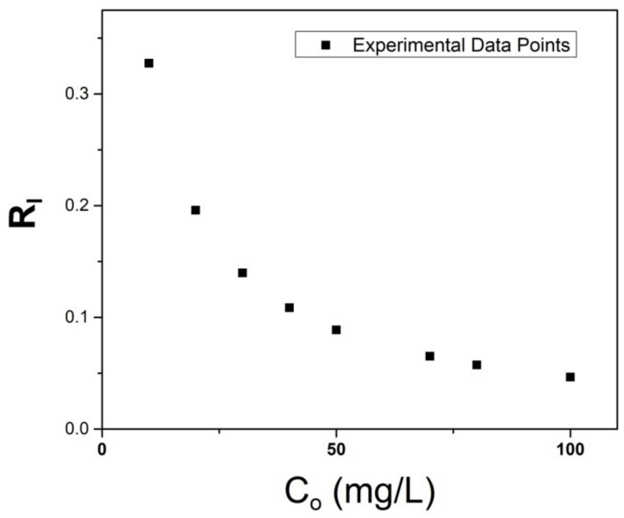

Supplementary Fig. S7: Dependence of R_l on intial concentration of Cd (II)

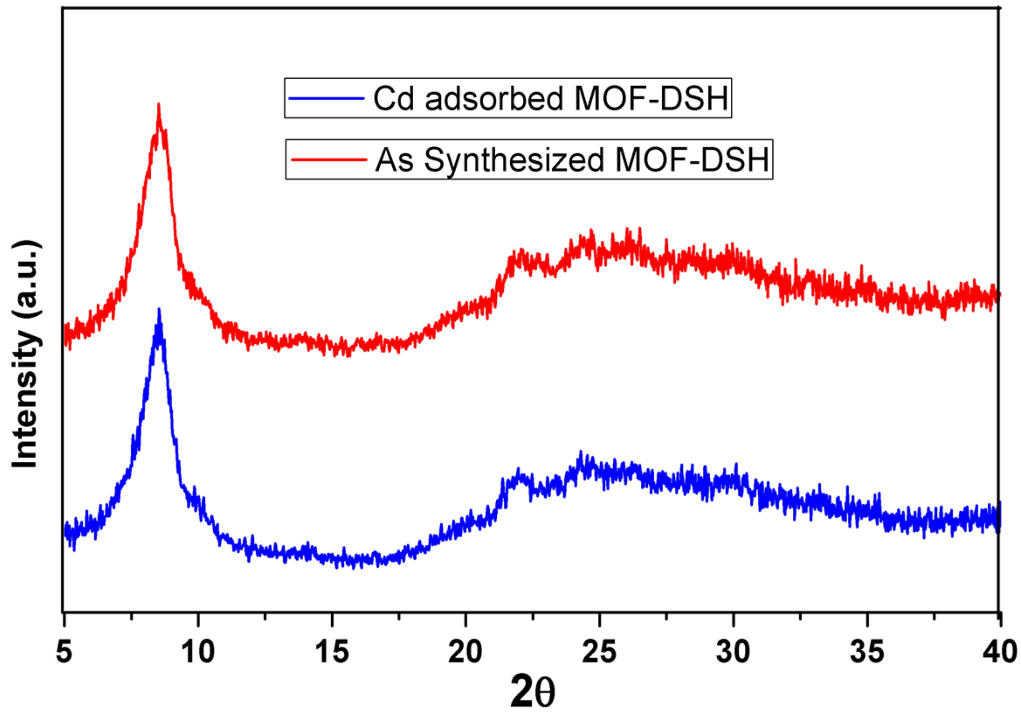

Supplementary Fig. S8: XRD patterns of MOF-DSH before and after Cd (II) adsorption

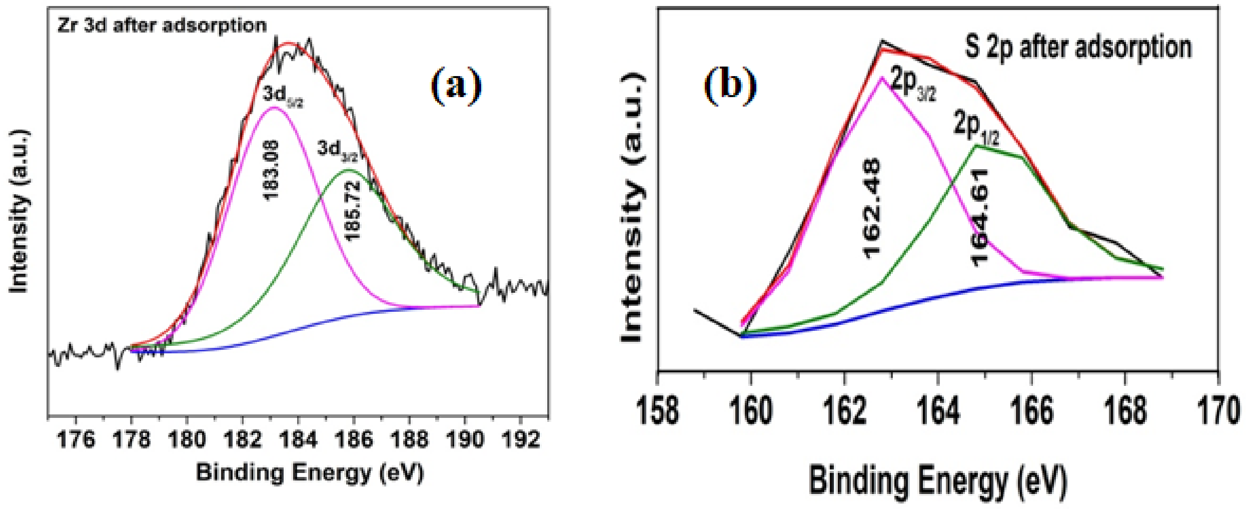

Supplementary Fig. S9: XPS spectrum of (a) Zr 3d after Cd adsorption; (b) S 2p after Cd (II) adsorption

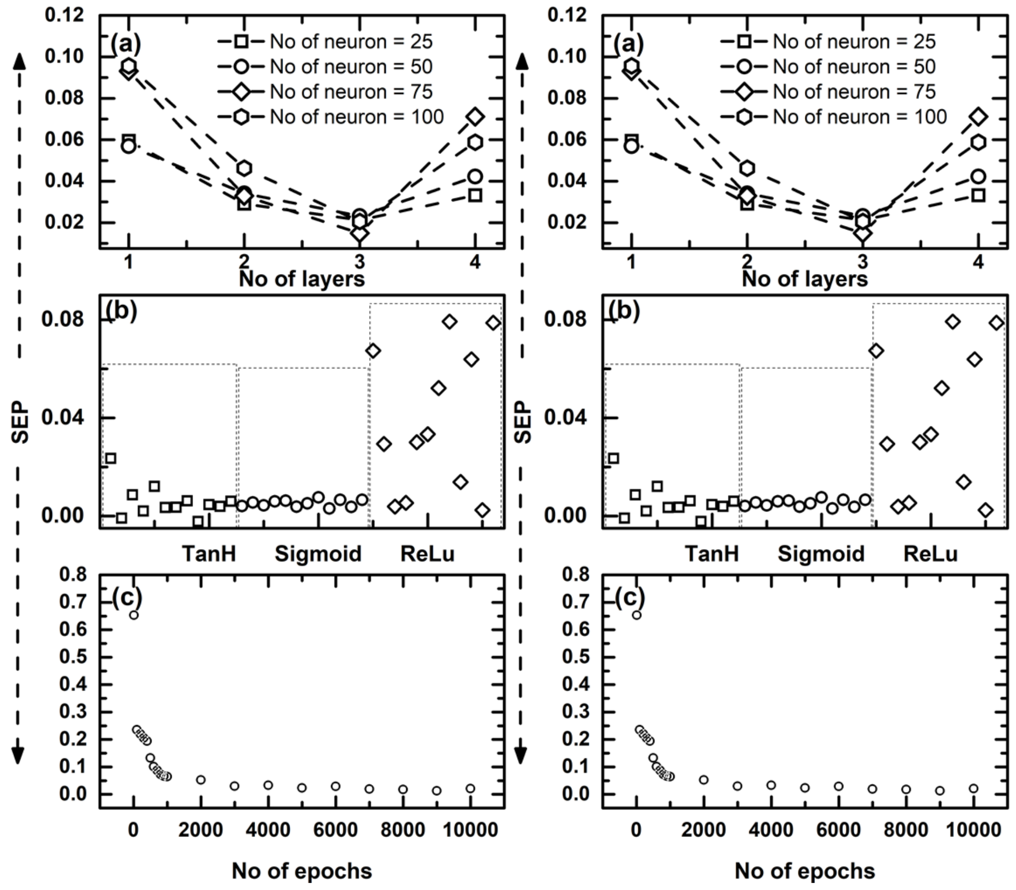

Supplementary Fig. S10: Optimization of the ANN architecture, variation of SEP values with (a) neuron number for different no. of hidden layers, (b) activation function for optimized ANN architecture and (c) different iteration no.

Supplementary Table S1. Adsorption capacity of MOF-DSH towards Cd (II) with change in pH

pH	Adsorption Capacity (mg/g)
2	3.3 ± 0.1
3	6.6 ± 0.15
4	36 ± 1.2
5	44 ± 2
6	45 ± 2
7	45 ± 1

Supplementary Table S2. Adsorption capacity of MOF-DSH with time variation

Time (minutes)	Adsorption Capacity (mg/g)
10	10 ± 2.1
30	16.6 ± 2.5
60	33.3 ± 2.2
120	43.3 ± 2.05
180	46.6 ± 2
240	48.3 ± 0.6
280	51.6 ± 1.8
340	53.3 ± 2.4
400	56.6 ± 1.1
1260	78.3 ± 1.5
1440	81.6 ± 0.5

Supplementary Table S3. Variation of R_l with change in initial concentration of Cd (II)

R_l	C_o (mg/L)
0.04645	100
0.057396	80
0.065061	70
0.088776	50

0.10856	40
0.139692	30
0.195858	20
0.327561	10

Supplementary Table S4. Removal (%) of different metal ions present together in aqueous solution using MOF-DSH

**Ion**	**Removal (%)**
K	2 ± 1
Ca	4 ± 1
Co	5 ± 1
Ni	4 ± 1
Sr	5 ± 1
Ag	4 ± 1
Cd	36 ± 2
Pb	37 ± 2

Supplementary Table S5. Configuration for optimizing the ANN structure in the present study

Total no. of neurons	No of hidden layers			
	1	2	3	4
25	3-25-1	3-20-5-1	3-12-8-5-1	3-12-8-3-2-1
50	3-50-1	3-40-10-1	3-25-15-10-1	3-20-15-10-5-1
75	3-75-1	3-50-25-1	3-50-20-5-1	3-40-20-10-5-1
100	3-100-1	3-80-20-1	3-50-30-20-1	3-50-30-15-5-1

References

1. Sanyal, K.; Kanrar, B.; Misra, N. L.; Czyzycki, M.; Migliori, A.; Karydas, A. G., A comparative study on the total reflection X-ray fluorescence determination of low Z elements using X-ray tube and synchrotron radiation as excitation sources. *X-Ray Spectrometry* **2017,** *46* (3), 164-170.
2. Wobrauschek, P.; Prost, J.; Ingerle, D.; Kregsamer, P.; Misra, N. L.; Streli, C., A novel vacuum spectrometer for total reflection x-ray fluorescence analysis with two exchangeable low power x-ray sources for the analysis of low, medium, and high Z elements in sequence. *Review of Scientific Instruments* **2015,** *86* (8), 083105.
3. Klockenkamper, R., *Total-Reflection X-Ray Fluorscence Analysis*. Wiley Interscience: 1997; Vol. 140.
4. Solé, V. A.; Papillon, E.; Cotte, M.; Walter, P.; Susini, J., A multiplatform code for the analysis of energy-dispersive X-ray fluorescence spectra. *Spectrochimica Acta Part B: Atomic Spectroscopy* **2007,** *62* (1), 63-68.
